# The beagle dog MicroRNA tissue atlas: identifying translatable biomarkers of organ toxicity

**DOI:** 10.1186/s12864-016-2958-x

**Published:** 2016-08-17

**Authors:** Erik M. Koenig, Craig Fisher, Hugues Bernard, Francis S. Wolenski, Joseph Gerrein, Mary Carsillo, Matt Gallacher, Aimy Tse, Rachel Peters, Aaron Smith, Alexa Meehan, Stephen Tirrell, Patrick Kirby

**Affiliations:** 1 Takeda Pharmaceuticals International Co., 40 Landsdowne Street, Cambridge, MA 02139 USA; 2 Eli Lilly and Company, 893 S. Delaware, Indianapolis, IN 46285 USA

## Abstract

**Background:**

MicroRNAs (miRNA) are varied in length, under 25 nucleotides, single-stranded noncoding RNA that regulate post-transcriptional gene expression via translational repression or mRNA degradation. Elevated levels of miRNAs can be detected in systemic circulation after tissue injury, suggesting that miRNAs are released following cellular damage. Because of their remarkable stability, ease of detection in biofluids, and tissue specific expression patterns, miRNAs have the potential to be specific biomarkers of organ injury. The identification of miRNA biomarkers requires a systematic approach: 1) determine the miRNA tissue expression profiles within a mammalian species via next generation sequencing; 2) identify enriched and/or specific miRNA expression within organs of toxicologic interest, and 3) in vivo validation with tissue-specific toxicants. While miRNA tissue expression has been reported in rodents and humans, little data exists on miRNA tissue expression in the dog, a relevant toxicology species. The generation and evaluation of the first dog miRNA tissue atlas is described here.

**Results:**

Analysis of 16 tissues from five male beagle dogs identified 106 tissue enriched miRNAs, 60 of which were highly enriched in a single organ, and thus may serve as biomarkers of organ injury. A proof of concept study in dogs dosed with hepatotoxicants evaluated a qPCR panel of 15 tissue enriched miRNAs specific to liver, heart, skeletal muscle, pancreas, testes, and brain. Dogs with elevated serum levels of miR-122 and miR-885 had a correlative increase of alanine aminotransferase, and microscopic analysis confirmed liver damage. Other non-liver enriched miRNAs included in the screening panel were unaffected. Eli Lilly authors created a complimentary Sprague Dawely rat miRNA tissue atlas and demonstrated increased pancreas enriched miRNA levels in circulation, following caerulein administration in rat and dog.

**Conclusion:**

The dog miRNA tissue atlas provides a resource for biomarker discovery and can be further mined with refinement of dog genome annotation. The 60 highly enriched tissue miRNAs identified within the dog miRNA tissue atlas could serve as diagnostic biomarkers and will require further validation by in vivo correlation to histopathology. Once validated, these tissue enriched miRNAs could be combined into a powerful qPCR screening panel to identify organ toxicity during early drug development.

**Electronic supplementary material:**

The online version of this article (doi:10.1186/s12864-016-2958-x) contains supplementary material, which is available to authorized users.

## Background

MicroRNAs (miRNA) are varied in length, under 25 nucleotides, single-stranded noncoding RNA that regulate post-transcriptional gene expression via translational repression or mRNA degradation [1]. miRNAs have properties which makes them ideal candidates for bio-fluid based biomarkers. miRNAs are detectable in a wide variety of biofluids [2–5], are stable in serum and can be quantified using sensitive and specific qPCR assays. miRNAs have been associated with and put forth as putative biomarkers of human disease, including hepatitis C (miR-122) [6], cardiovascular diseases (miR-192) [7] and various types of cancers [8].

Many miRNAs are highly conserved among mammalian species and demonstrate tissue-specific expression [1, 9–14]. In humans, there are approximately 2600 annotated human miRNAs in miRBase (v. 21) [15]. Studies comparing expression among vertebrates (zebrafish, chickens, and mice) have demonstrated that miRNA expression is not strictly conserved [11]. Not all human miRNAs are detected in other mammalian species [10, 14, 16]. However, there are examples of validated biomarkers such as miR-122, which is detectable in the blood after liver injury in mice, rats, dogs and humans [9, 17–20]. Thus, miRNA biomarkers have great potential as translatable tools to monitor for organ specific injury throughout all phases of drug safety assessment.

The identification of novel miRNA biomarkers requires a systematic approach to: 1) determine the miRNA tissue expression profiles within a mammalian species via next generation sequencing (miR-seq); 2) identify enriched miRNA expression within organs of toxicologic interest; and 3) validate in vivo using select toxicants. miRNA expression atlases and candidate biomarkers of organ specific injury have been investigated to varying degrees in rodents and humans [5, 12, 21]. Dogs are a relevant preclinical species that are used in nonclinical safety assessments [22, 23]. However, little is known about dog miRNA expression or annotation of sequences.

This work describes an miRNA atlas of 16 tissues from male Marshall Beagle dogs that was compiled using miR-seq. In the miRNA dog tissue atlas presented, 106 tissue enriched miRNAs were identified, 66 of which demonstrated a high level of enrichment in different tissues. Comparison of the dog atlas to currently available literature suggests that most highly enriched miRNAs are conserved across the target organs. The most unique miRNAs identified in the dog tissue atlas were found in the central nervous system, which is consistent with other species [5, 24]. Quantitative reverse transcription PCR (Q-RT-PCR) of tissue extracts confirmed the enrichment of most miRNAs identified by miR-seq analysis. Finally, the specificity of the candidate miRNA biomarkers was demonstrated through proof of concept (POC) studies with established liver toxicants.

In parallel to this effort, Eli Lilly authors have constructed a rat miRNA tissue atlas using miR-seq in 5 male and 5 female Sprague Dawley rats and 21 and 23 organs, respectively. Publication of both miRNA tissue atlases will provide an invaluable resource for the characterization of potential biomarkers of organ injury in the two most common nonclinical species used in drug development safety assessment.

The ultimate objective of this collaboration will be to assemble a serum based Q-RT-PCR screening panel of validated miRNA biomarkers of organ injury. The basic miRNA panels described herein and in the complimentary rat miRNA tissue atlas manuscript may be valuable tools in the early stages of drug development to understand test article-related target organ profiles.

## Methods

### Tissue sample collection

Sixteen different tissues were harvested from five 10-month old male Marshall Beagle dogs at Bioreclamation, LLC including: liver, heart, testis, lung, skeletal muscle (quadriceps), kidney, thymus, brain, sciatic nerve, pancreas, small intestine (duodenum, jejunum, and ileum), colon, bone marrow, and plasma. In order to minimize the contribution of blood cells to miRNA expression detected in individual organs, dogs were perfused with phosphate buffered saline prior to collection of organs analyzed in the dog tissue atlas. Tissue samples were placed in RNAlater® solution (Life Technologies), snap frozen in liquid nitrogen, and then stored at -80 °C until RNA isolation and miR-seq analysis.

### RNA isolation

For tissues, samples were homogenized with lysis buffer (RLT [Qiagen] + 1 %-β mercaptoethanol) using a rotor-stator homogenizer. RNA was extraced from tissue homogenate with KingFisher™ Pure RNA Tissue Kit (Thermo Scientific). Briefly, tissue homogenates were combined with magnetic beads and ethanol, and loaded onto a KingFisher Magnetic Particle Processor (Thermo Scientific). Samples were DNase-treated, washed, and eluted in RNase-free water. Due to the low level of total RNA isolated from dog sciatic nerve, the five individual samples were pooled and split into three sample replicates for analysis. RNA isolation of the bone marrow was performed with 3-fold volume of TriReagent LS (Life Technologies). Additional details are in the Additional file 1.

### Assessment of RNA purity, quantity, and integrity

The purity and quantity of total RNA samples were determined by absorbance readings at 260 and 280 nm using a NanoDrop ND-1000 UV-Vis spectrophotometer (Thermo Scientific). The integrity of total RNA was assessed by capillary electrophoresis using an Agilent Bioanalyzer 2100 (Agilent).

### miR-seq annotation and sample analysis

Dog miRNAs were aligned to the canine genome identified by the annotation prefix *canis familiaris* annotation (cfa). The annotation of mature miRNAs within miRBase was much smaller for dog (453 miRNAs) than for rat (765) or human (2588). Given the hypothesis that most miRNAs are conserved across mammalian species, a more comprehensive annotation of the dog miRNA tissue atlas was conducted using annotation from dog, rat, and human mature miRNA sequences. This was accomplished in four sequential steps: 1) identification of mature miRNA sequences for dog, rat, and human (*n* = 3896) using miRBase v.21; 2) consolidation of mature miRNA by eliminating sequences that were conserved across all three species (*n* = 3355); 3) alignment of sequences from the dog genome using Omicsoft Sequence Aligner (OSA) v4, and retained sequences that aligned with zero mismatches (*n* = 1087); and 4) merging of miRNAs that aligned to approximately the same location. This process resulted in the annotation of 857 miRNAs in the dog genome, including 314 putative dog miRNAs that were annotated using rat and/or human miRNA sequences which were not present in the current dog miRNA genome.

Total RNA from dog tissue samples were analyzed using miR-seq. A TruSeq Small RNA Library Kit (Illumina) was used for library construction of dog tissue RNA. Sequencing was performed on the GAIIx (Illumina) at 36 base pair read length and targeting 12 million reads per sample. Adaptor sequences were clipped and OSA v4 (http://omicsoft.com/osa) was used to align the reads to the dog genome (CanFam3.1) [25] allowing zero mismatches and excluding any reads that aligned to greater than 10 genomic locations. Expression levels were quantified using bedtools (https://github.com/arq5x/bedtools2). Aligned reads were counted in miRNA expression only if they overlapped with at least 70 % of known or putative miRNA loci. Additional details of analyses are described in the Additional file 1.

Hereafter, we define two terms to describe the enrichment status of miRNA: *tissue enriched* (TE) and *highly tissue enriched* (HTE). Briefly, an miRNA was considered to be TE if the fold-change level within the tissue of interest was > 5, and the FDR-corrected Wilcoxon Rank-Sum *p*-value was < 0.05 [26]. To be classified as HTE, the miRNA median expression required > 5 fold higher expressed compared to the maximum expression value observed in any other tissue analyzed and was only detected in ≤ 2 tissues within the atlas. Details of the criteria used for these terms are described in the Additional file 1.

### miRNA atlas verification

Q-RT-PCR analysis was conducted to verify the expression levels of candidate miRNAs identified via miR-seq. Briefly, total RNA was reverse transcribed using the Applied Biosystems TaqMan MicroRNA RT Kit (Life Technologies) according to manufacturer instructions. For each PCR reaction, cDNA was mixed with Applied Biosystems TaqMan Universal PCR Master Mix, No AmpErase® UNG (Life Technologies). All amplifications were performed in triplicate using a 7900HT Fast Real-Time PCR System (Applied Biosystems). Technical replicate threshold cycle (C_t_) values were averaged for each sample. C_t_ values greater than 38 were removed from the analysis because they were within a 10-fold level of the final cycle and were deemed less reliable or not expressed. Relative miRNA expression quantification was derived using the ΔCt method [27]. Additional experimental details are described in the Additional file 1.

### Atlas utilization in dog toxicology studies

Naïve male beagle dogs (approximately 10 months old) were acquired from Marshall BioResources. Animals were housed in a facility accredited by the Association for Assessment and Accreditation of Laboratory Animal Care International. At the end of study, liver samples were harvested and placed in neutral buffered formalin for a minimum of 24 h before trimming, embedding, and histopathologic processing. Slides for histopathologic assessment were prepared using hematoxylin and eosin staining. Histopathologic assessment of liver sections was conducted by a board certified veterinary pathologist.

### Assessment of a 5 miRNA biomarker screening panel in a 7-day repeat-dose study with Compound X

Three male dogs per group were administered either vehicle (10 % hydroxypropyl-beta-cyclodextrin [HP-β-CD] + 3.5 % NaHCO_3_) or Compound X at 500 mg/kg via oral gavage twice daily for seven consecutive days. Animals were fasted overnight prior to the end of study on Day 8 (24 h after the last dose). Liver tissues were harvested for histopathologic assessment and serum was collected for serum chemistry and Q-RT-PCR analysis using a 5 miRNA biomarker screening panel. The miRNAs evaluated were cfa-miR-122 and cfa-miR-885 (both considered to be highly liver enriched) as well as putative negative controls for heart (cfa-miR-1), pancreas (cfa-miR-216a), and muscle (cfa-miR-133a).

### Assessment of a 22 miRNA biomarker screening panel in a 14-day repeat-dose study with Compound Y

Six male dogs per group were administered either vehicle (0.5 % methylcellulose) or 600 mg/kg of Compound Y via oral gavage daily for 14 days. Blood samples were collected on Days 1, 7, and 14 (at predose, and 24 h postdose) for serum chemistry and Q-RT-PCR analysis of 22 miRNAs. The miRNAs evaluated were cfa-miR-122 and cfa-miR-885, and an extended panel of non-liver enriched miRNAs for heart/muscle (cfa-miR-1, -133a/b, and -208b), heart (cfa-miR-499), muscle (cfa-miR-206), brain and sciatic nerve (cfa-miR-212 and -432), testis (cfa-miR-34b/c), pancreas (cfa-miR-216a/b), putative liver (cfa-miR-21 and -192) and ubiquitous controls (cfa-miR-16, -29a, and -186). Dogs were euthanized on Day 15 (24 h after the last dose) and liver tissue was harvested for microscopic assessment.

## Results and discussion

### Atlas tissue, RNA, and sequencing quality

This work describes the first comprehensive miRNA atlas of 16 tissues from male beagle dogs that was generated using miR-seq. Similar miRNA tissue atlases exist for other toxicologically relevant nonclinical species, such as the rat and monkey [5, 28]. Collectively, these atlases may contribute to the identification and monitorability of organ specific toxicities and provide an additional tool in the drug development process.

**Fig. 1:**
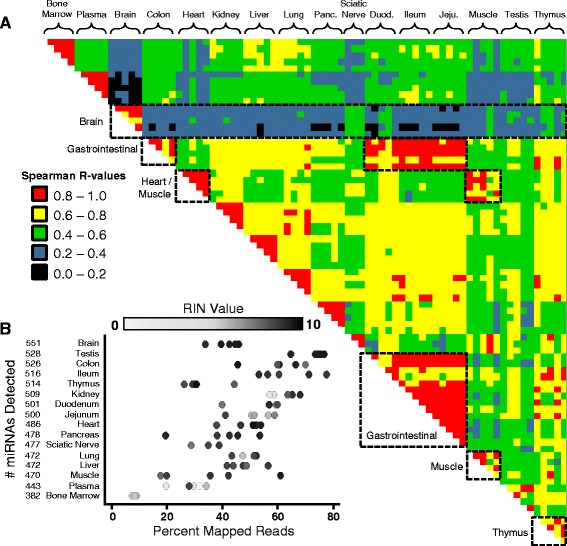
miRNA correlation between tissue samples. **a** Spearman’s rank correlation within tissue type and across all tissues in the dog miRNA tissue atlas with segmented R-values ranked from highest to lowest. Analysis of miRNA tissue altas miR-seq results identified unique expression patterns observed in the dog brain, similarity in miRNA expression between muscle and heart and in gastrointestinal tissues, as well as intertissue heterogeneity in the thymus. **b** Percent mapped reads per individual tissue sample and their corresponding RIN values ranging from zero (*white*) to ten (*black*). Tissue columns are sorted least to most abundant median detected miRNAs by tissue

Data from five samples per tissue type were included in miR-seq analysis to generate the dog miRNA tissue atlas with the following exceptions: only 4 of 5 colon samples and 3 sciatic nerve miRNA samples were included in downstream enrichment analysis. The 3 sciatic nerve samples comprised 2 individual samples and 3 samples were pooled due to low RNA yields. Quality assessments used for sample analysis included Spearman’s (Rank *r*^2^) correlation of miRNA expression across all tissue samples (Fig. 1a), total tissue RNA (RNA integrity number [RIN]) the number of miRNAs, and the percent mapped reads in each tissue sample (Fig. 1b). Based on Spearman’s correlation, the majority of tissue expression profiles demonstrated good reproducibility between tissue samples of the same organ (average *r*^2^ > 0.85) with the exception of muscle, duodenum, testis, and thymus (Fig. 1a). Tissues with similar miRNA expression patterns between organs included heart and muscle (average *r*^2^ = 0.8) and tissues of the upper and lower gastrointestinal (GI) tract (duodenum, jejunum, ileum, and colon; average *r*^2^ = 0.9). In contrast, the brain demonstrated the most distinct miRNA expression profile (average *r*^2^ = 0.3) when compared to all other tissues examined.

### miRNA sequencing: tissue enrichment analysis

#### miRNA alignment, annotation, and quantification

**Fig. 2:**
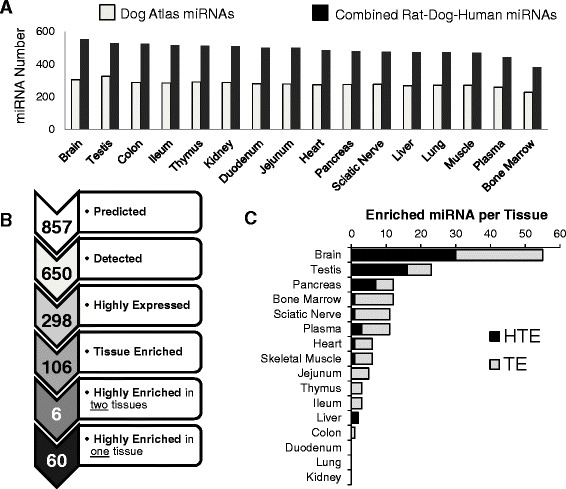
Dog atlas miRNAs detected and enriched by tissue type. **a** Annotated dog miRNAs identified in this atlas (*gray bars*). The number of annotated miRNAs increases when using dog, rat, and human annotation (*black bars*). **b** The tissue histogram of the number of enriched miRNAs, sorted from lowest to highest, by tissue and enrichment level: TE (*gray*), HTE in 2 tissues (*dark gray*), and HTE in 1 tissue (*black*). **c** Summary of the HE and HTE miRNAs identified in all 16 dog tissues

There are 453 annotated mature miRNA sequences for the dog in miRBase (v.21), far less than for rat (765) or human (2588). To increase the numbers of known miRNAs, a *de novo* annotation of dog miRNAs was performed using known rat and human sequences as guides (Fig. 2a). Sequence alignment with the combined rat, dog, and human miRNA annotation resulted in the identification of 857 putative homologous miRNAs within the dog genome. Of these, 650 miRNAs (355 from dog and 295 from rat and/or human) were detected by miR-seq and aligned to the genome of dogs, and 106 potential miRNA biomarkers were identified (Fig. 2b). A comprehensive list of the 106 potential miRNA biomarkers and the tissues in which they were enriched is presented in Fig. 2c. The similarity in sequences between the dog miR-seq data and the 295 miRNAs annotated using rat/human miRNA homologs strongly suggests that these miRNAs, while not currently annotated in the dog genome, are likely well conserved in the dog as well as in rat and human. Additionally, 20 of the 66 highly tissue expressed miRNAs were not annotated in the dog and instead were identified through rat/human homologs.

### miRNA enrichment

**Fig. 3:**
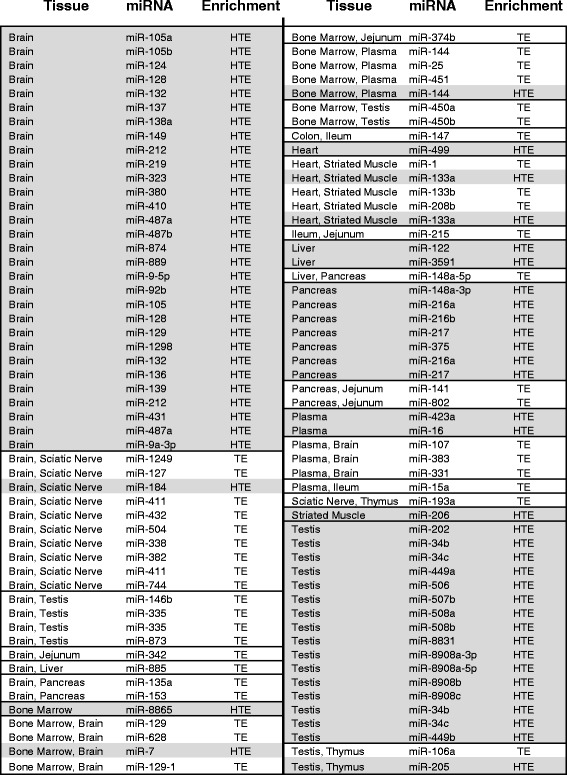
List of 106 enriched dog miRNAs by tissue. miRNAs are annotated as tissue enriched (TE) or highly tissue enriched (HTE). Only miRNA stem loop names are shown for sake of ease

This study identified 298 miRNAs with normalized reads per million (RPM) greater than 100 in at least one tissue. Of these, 214 miRNAs were annotated dog miRNAs and 84 were identified using rat and/or human sequences. A total of 106 dog miRNA sequences were TE, 66 miRNAs were HTE and of these, 60 were enriched in only one tissue while the remaining 6 were enriched in two tissues (Fig. 3). Additional information about specific mature miRNA homologs in rats and humans is presented in Additional file 2: Figure S1. The top 20 TE/HTE miRNAs from the dog atlas (Additional file 3: Figure S2) and top 10 expressed miRNAs per tissue (Additional file 4: Figure S3) are also presented.

Brain tissue had the greatest number of enriched miRNAs (55), including 30 HTE and 25 TE miRNAs (Fig. 3). This is consistent with previous findings that approximately 70 % of miRNAs are expressed in the brain [29]. Rat studies identified numerous enriched brain miRNA, including miR-9, -124, -128, -184, and -219 [5, 9, 30]. There are 14 known enriched miRNAs in the human brain [29]. Four dog brain HTE miRNAs (cfa-miR-9, -124, -128, and -219) share sequence homology with rat and human, and are cross-species biomarker candidates for brain injury. Of these, miR-9 was among the highest expressed miRNAs in the dog atlas (Additional file 3: Figure S2).

The testes had the second largest number of tissue enriched miRNAs (23), including 16 HTE and 7 TE (Fig. 3). Interestingly, 10 testes HTE miRNAs were among the most highly expressed miRNAs across all tissues, including the two highest level of expression miRNAs (cfa-miR-508b and -202) observed within the entire dog miRNA tissue atlas (Additional file 3: Figure S2). Except for miR-34b/c, which has a role germ cell maturation in murine testes [31, 32], none of these miRNAs have reported associations with testis. Testes enriched cfa-miR-34b/c, and the other testes HTE dog miRNA identified here may be invaluable as biomarkers for an organ that presently lacks a definitive diagnostic test for tissue injury.

There were 12 enriched miRNAs observed in the pancreas (7 HTE and 5 TE) (Fig. 3). Of these, only cfa-miR-216a correlated with observed expression in both mouse and rat pancreas [33]. A recent rat study demonstrated that plasma levels of miR-216a and miR-216b increased after pancreatic injury [34]. The pancreatic HTE cfa-miR-217-3p had an annotated homolog in rat but not human (Additional file 5: Figure S4). This novel finding suggests that human miR-217 should in fact be two separate mature miRNA sequences: miR-217-5p and miR-217-3p.

The two liver HTE miRNAs in dogs were miR-122 and the reverse complement miR-3591 (Fig. 3). In the literature, miR-122 is liver specific for mice, rats, and humans and has been used as a biomarker for liver injury [5, 10, 12]. miR-855 is a liver TE miRNA reported to be significantly elevated in sera from patients with liver disease [35]. Others have reported miR-192 as a liver specific biomarker in multiple species [9, 20]. In contrast, the findings presented in this dog atlas show that cfa-miR-192 was expressed at relatively high levels (>650 RPM) in all tissues. Because this miRNA did not meet the TE miRNA criteria, it does not appear to be a suitable biomarker of liver injury in the dog.

A single HTE miRNA was identified in the dog heart (cfa-miR-499) and in skeletal muscle (cfa-miR-206), and both tissues had the same 5 TE miRNAs (cfa-miR-1, -133a-5p, -133a-3p, -133b, and -208) (Fig. 3). Enrichment of miR-499 in the heart has been demonstrated in rat, monkey, and human [36, 37]. Likewise, miR-206 is enriched in the skeletal muscle from rodents and humans [38]. The overlap of heart and muscle miRNA expression is supported by the Spearman’s correlation (Fig. 1) and is consistent with the literature [39]. The family of ‘myomiRs’ (miR-1, -133a, -133b, and -208) is known to be expressed in both cardiac and muscle tissues [39, 40], and was enriched in the myocardium of rats, dogs, and monkeys [41].

Analysis of dog plasma identified 3 HTE (cfa-miR-423a, -144-5p, and -16-3p) and 8 TE miRNAs (Fig. 3). Of the plasma HTE miRNAs, only miR-144 is known to be blood cell specific [5] and is involved in erythrocyte homeostasis [42]. Analysis of dog bone marrow identified 1 HTE miRNA (miR-8865) and 11 TE miRNAs. Only TE miRNAs were identified in the thymus (3) and GI tract (7). There were no HTE or TE miRNAs found in either the dog lung, kidney or duodenum.

There were 3 dog miRNAs (cfa-miR-16, -29a, and -186) with ubiquitous expression across all tissues. miR-16 is regarded as a stably expressed miRNA in human and has been used as a reference miRNA for normalization of serum miRNA biomarkers for breast, prostate, and colorectal cancers [43–45]. Both miR-29a and miR-186 are some of the most frequently detected miRNAs in plasma and serum [46]. Likewise, all three miRNAs were abundantly detected, have low CV < 10 %, and standard deviation of 1 or below when compared across all tissues analyzed in the dog miRNA atlas. While these miRNAs were used to normalize miR-seq validation by qPCR data, they could not be used in POC studies because their serum levels unexpectedly differed between control animals and dosed animals.

### Atlas verification

Tissue samples were analyzed by Q-RT-PCR to confirm the expression levels of miRNAs identified as HTE or TE by miR-seq analysis. A total of 22 miRNAs (Additional file 6: Figure S5) were selected for qPCR validation including the following 14 biomarker candidates of organ toxicity: liver (cfa-miR-122 and -885), pancreas (cfa-miR-216a/b); heart (cfa-miR-499); muscle (cfa-miR-206); heart/muscle (cfa-miR-1, -133a/b, and -208); testis (cfa-miR-34b/c); and brain and sciatic nervous tissues (cfa-miR-212, -432, and -885), and 5 miRNAs reported in the literature (cfa-miR-21, -192, -193a/b, and -200). For Q-RT-PCR normalization controls, 3 miRNAs identified in the dog atlas (cfa-miR-16, -29a, and -186) were selected on the basis that they are ubiquitously expressed at similar levels in all tissues examined.

**Fig. 4:**
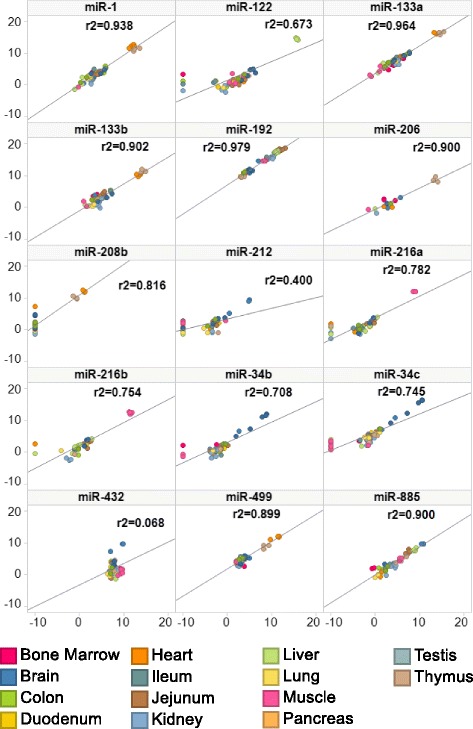
qPCR verification of atlas miRNA-seq of 15 potential biomarkers of organ toxicity. Q-RT-PCR values represent the normalized log2 expression values (y-axis) and normalized log2 values miR-seq (x-axis) for individual animal tissue samples tested. Correlation analysis (linear regression) demonstrated agreement of tissue enriched miRNA across both platforms

Eleven of 15 miRNAs examined by miR-seq and Q-RT-PCR miRNA platforms demonstrated comparable expression levels of individual miRNAs selected for verification with *r*^2^ values > 0.6 (Fig. 4). The exceptions (cfa-miR-432, -499, and -212) could not be differentiated between tissues analyzed by Q-RT-PCR. The 3 highly expressed ubiquitous miRNAs demonstrated good precision, as measured by the coefficient of variance (%CV = standard deviation/mean), for all tissues in both detection platforms; miRNA-SEQ (cfa-miR-16 [4.9 %], -29a [4.7 %], and -186 [1.8 %]) and Q-RT-PCR (cfa-miR-16 [4.1 %], -29a [4.6 %], and -186 [2.7 %]).

### Highest expressed miRNA in the kidney

Although enriched miRNAs were not detected in the dog kidney, this is a unique organ because urine can be evaluated for biomarkers. Thus, the highest expressed kidney miRNAs may possess the greatest potential as urinary biomarkers of kidney injury. Circulating blood miRNAs are not thought to pass through the kidney into the urine intact [3]. High expression in dogs was observed for cfa-miR-10a, -10b, -22, -181a, -191, -192, and -378 (Additional file 4: Figure S3). There was a strong overlap between the miRNA identified here in the dog whole kidney and the highest expressed miRNA identified by Ichii et al. in the dog cortex (7/10) and medulla (8/10) (Additional file 7: Figure S6) [47]. Kidney enriched miR-10a and miR-192 were previously identified as potential circulating biomarkers of renal injury in rats [48], however miR-192 had high expression in multiple dog tissues and was not identified as kidney enriched in the current study. Other miRNAs have been implicated in various kidney diseases, including in renal fibrosis (miR-22) [49], nephritic syndrome (miR-181a) [50], and renal cell carcinoma (miR-378) [51]. Levels of miR-191 and miR-378 were elevated in the urine of rats dosed with nephrotoxicants [52, 53]. It remains to be determined whether any of these highly expressed renal miRNAs can be detected in the urine and outperform standard protein-based biomarkers of renal injury.

### Liver safety biomarkers POC studies

One concern for using miRNAs as circulating biomarkers is how to normalize across samples [54]. While studies have investigated the use of spiking samples with miRNA from exogenous sources such as *Arabidopsis* or *C. elegans* [55, 56] or normalization by serum or plasma volume, miRNA in the current study were normalized using total counts. The expression patterns of miRNA in plasma versus serum are similar [46, 56, 57]. Due to sample availability only serum was analyzed in the dog POC studies.

#### Assessment of a 5 miRNA biomarker screening panel after 7 days of dosing with Compound X

**Fig. 5:**
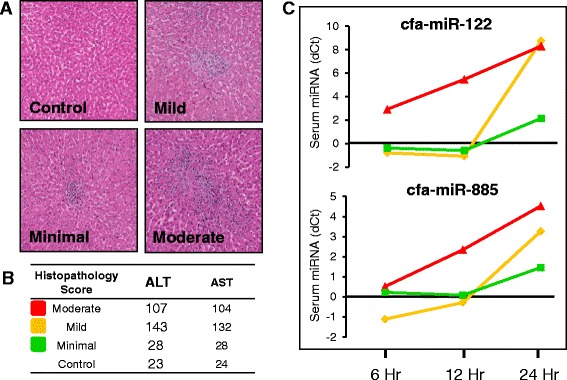
POC Compound X histopathology review and candidate safety biomarker assessment. **a** Representative hematoxylin and eosin stained liver histopathology sections. Liver damage severity was scored by histopathologic review as Minimal, Mild, or Moderate. **b** Serum levels of ALT and AST in animals that received Compound X or vehicle control. The colored boxes on the left correspond to the severity of liver injury. **c** Serum levels of miR-122 and miR-885 in animals dosed with Compound X compared to vehicle. Samples were collected at the indicated time points on Day 7 of dosing. The line color corresponds to the severity of damage

Three dogs were dosed twice daily for seven days with 500 mg/kg of Compound X or vehicle. At 24 h post-Day 7 dosing, two dogs demonstrated mild to moderate liver necrosis (Fig. 5a) and had an approximately 5-fold elevation in serum alanine aminotransferase (ALT) and aspartate aminotransferase (AST) which correlated with mild to moderate liver necrosis (Fig. 5b). Analysis of serum miRNA from these two dogs showed the highest elevation (ΔCt) values in cfa-miR-122 and cfa-miR-855 compared to control animals (Fig. 5c). Histopathological analysis did not identify microscopic changes in heart, muscle and pancreas in dogs treated with Compound X, and the levels of non-liver enriched miRNAs (cfa-miR-1, -133 and -216) did not significantly change when compared to control dogs (data not shown). Taken together, these results highlight the specificity of miR-122 and miR-885 for compound-induced liver injury.

#### Assessment of a 20 miRNA biomarker screening panel after 14 days of dosing with Compound Y

Six dogs were dosed daily with 600 mg/kg of Compound Y or vehicle control for 14 days. Serum samples collected on Days 1, 7, and 14 (predose and 24 h postdose) were analyzed for ALT and AST levels. Serum was also analyzed by Q-RT-PCR for a panel of 20 tissue enriched and potential miRNA biomarkers, including those identified for liver (cfa-miR-122 and -885), heart/muscle (cfa-miR-1, -133, and -206), testis (miR-34b/c), pancreas (cfa-miR-216), brain (cfa-miR-212), and ubiquitously expressed cfa-miR-193b.

**Fig. 6:**
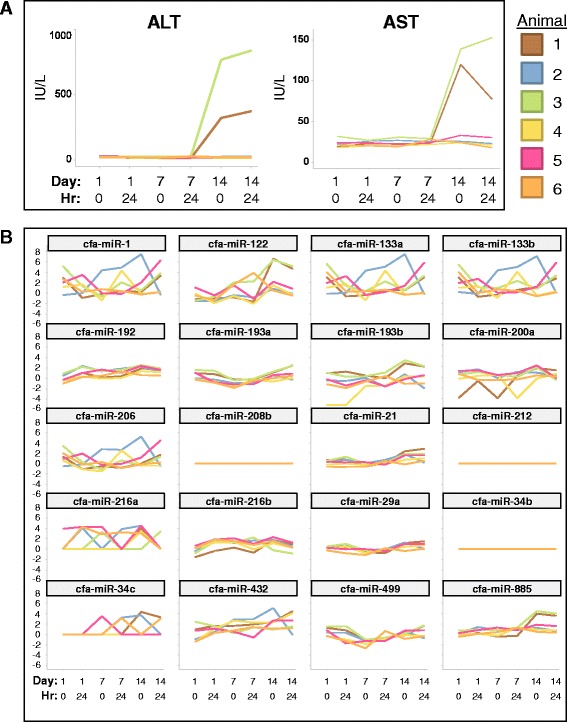
POC Compound Y miRNA safety biomarker panel assessment. **a** Serum levels of ALT and AST in animals that received Compound Y or vehicle control. Serum was collected on Days 1, 7 and 14 at pre-dose and 24 h post-dose. The colored boxes on the right correspond to the individual dog identification numbers. **b** Panel of candidate safety miRNA biomarkers utilized in the Compound Y POC study. Q-RT-PCR ΔCt values (y-axis) line plot per animal for duration of Day 1, 7, and 14 treatment samples tested highlights the elevation of both liver enriched miRNAs (miR-122 and miR-885) and ubiquitously expressed miR-193 in the 2 dogs with elevated ALT and AST. Non-liver enriched miRNAs were not elevated or were found to be in the noise or below the lower limit of Q-RT-PCR detection

Elevations in liver enriched miR-122 and miR-885 correlated with increases in ALT and AST (Fig. 6a and b) and mild to moderate hepatocellular necrosis was observed in 2 of 6 animals (Dogs 1 and 3 on Day 14 [data not shown]). Dogs 1 and 3 had high correlation between elevations in serum miR-122 and ALT (*r*^2^ = 0.79) and AST (*r*^2^ = 0.90) levels while elevations in serum miR-885 were higher ALT (*r*^2^ = 0.87) and AST (*r*^2^ = 0.97). Additionally, Dog 3 demonstrated elevated miR-122 on Day 7 at the 24 h time point while ALT remained at baseline levels. The four other animals dosed with Compound Y did not demonstrate test article-related changes in serum chemistry, miRNA or liver histopathology. Elevation of serum cfa-miR-193b in the two dogs with liver injury (Dogs 1 and 3) was likely due to the ubiquitous expression of the miRNA in the dog, including the liver. The transient serum elevations of heart/muscle TE miRNAs (cfa-miR-1 and -133) and muscle HTE miRNA (cfa-miR-206) were not correlated with microscopic findings (data not shown) and may be due to injury during animal handling. The remaining non-liver miRNAs were not detected in any serum samples.

Elevation of miR-122 in the plasma/serum after administration of hepatotoxic compounds in rats [58, 59] or in various human disease conditions [19, 60] is well established. The results from these two POC studies represent the first published data demonstrating the utility of miR-122 and miR-885 as potential biomarkers of liver injury in the dog. Levels of miR-122 increased in correlation with minimal liver injury in the absence of ALT elevations, and thus may have superior sensitivity than standard liver function tests. This is consistent with a previous rat study that detected an increase in miR-122 levels prior to ALT elevation [58]. While the sensitivity of miR-122 versus ALT has not been definitively determined in any species, these collective studies indicate that miR-122 is a translatable diagnostic to detect liver injury [61].

## Conclusion

miRNAs hold promise as circulating biomarkers of organ specific injury. While recent studies focused on miRNA tissue expression in rodent species and humans, there is a significant lack of miRNA data available for dogs. The beagle dog is an important nonclinical species for assessment of human drug safety. Identification of miRNA biomarkers of tissue specific injury in the dog may improve prediction and monitorability of potentially adverse effects of compounds intended for human use. This miRNA tissue atlas may also serve as a reference for the identification of novel biomarkers of organ damage in nonclinical species which are translatable to patients in the clinic. Analysis of the dog miRNA tissue atlas identified 66 HTE miRNAs which offer a starting point for exploratory pre-clinical safety monitoring, further proof of concept and biomarker validation studies beyond the liver biomarker potential described herein.

The dog miRNA annotation has been expanded through the identification of homologs to rat and human miRNAs in the dog genome which demonstrate a high level of sequence homology and similar levels of tissue expression among rats and humans. There are likely to be additional miRNAs in dogs that might have been tissue enriched but were not annotated. As the quality of annotation for dog miRNA improves, so will the resolution and quality of the dog miRNA database. The expression data may be re-evaluated at a later date to see if previously unannotated miRNA sequences can be identified. Conversely, information gathered from the dog miRNA atlas can be used to fill in gaps in the human annotation, as in the case with miR-217. The data generated for the dog miRNA tissue atlas is publically available for further analysis (The accession number is: GSE83278).

In collaboration with Eli Lilly, co-analyses of the dog and rat miRNA sequencing data determined which tissue enriched miRNAs were conserved between the two species. The next phase of the atlas will be to further demonstrate in vivo the correlation of tissue enriched miRNA to tissue specific injury. Subsequent animal studies are necessary to determine the sensitivity and specificity of the miRNA qPCR panel. Candidate biomarkers may eventually be used as an early readout of tissue injury in drug development.

## Abbreviations

ΔCt, change in threshold cycle; ALT, alanine aminotransferase; AST, aspartate aminotransferase; cDNA, complementary deoxyribonucleic acid; cfa, canis familiaris annotation; Ct, threshold cycle; CV, coefficient of variation; FDR, false discovery rate; GI, gastrointestinal; HP-β-CD, hydroxypropyl-beta-cyclodextrin; HTE, highly tissue enriched; miRNA was considered HTE if the miRNA median expression required > 5 fold higher expressed compared to the maximum expression value observed in any other tissue analyzed and was only detected in ≤ 2 tissues within the atlas; miRNA, microRNA; mRNA, messenger ribonucleic acid; OSA, Omicsoft Sequence Aligner; PCR, polymerase chain reaction; POC, proof of concept; qPCR, see Q-RT-PCR; Q-RT-PCR, quantitative reverse transcription polymerase chain reaction; RIN, ribonucleic acid integrity number; RNA, ribonucleic acid; RPM, reads per million; TE, tissue enriched; miRNA was considered to be TE if the fold-change levels within the tissue of interest was > 5, and the FDR-corrected Wilcoxon Rank-Sum *p*-value was < 0.05

## Additional files

Additional file 1: Supplementary Methods. (DOCX 18 kb) Additional file 2: Figure S1. List of 106 enriched miRNAs by tissue. miRNAs are annotated as tissue enriched (TE) or highly tissue enriched (HTE). The family of miRNA and mature miRNAs in dog, human and rat are given. (PDF 55 kb) Additional file 3: Figure S2. Top 20 enriched miRNAs. 10 of top 20 are testis. (PDF 38 kb) Additional file 4: Figure S3. Top Ten Expressed (log10 RPM values) miRNA per tissue type. The list is sorted by dog miRNA tissue atlas enrichment and prevalence of top 10 expressed miRNAs across the atlas tissues. (PDF 45 kb) Additional file 5: Figure S4. Benefit and need for enhanced annotations for miRNA studies. (A) Pancreas enriched miR-217-3p (rno-miR-217-3p AUCAGUUCCUAAUGCAUUGCCU) identified in the dog miRNA tissue atlas was found as a novel un-annotated human miRNA. It is conserved in rats, dogs, and humans, but is only annotated in miRBase as rat. (B) There is evidence of un-annotated human reads aligning and expressed in human expression profile experiments on the miRBase website. This study suggests human miRBase annotation should reflect 2 mature microRNA sequences, miR-3p and -5p. (PDF 64 kb) Additional file 6: Figure S5. Q-RT-PCR assay list. (PDF 39 kb) Additional file 7: Figure S6. Top 20 expressed kidney miRNA. Top 20 expressed kidney miRNA found in the dog miRNA tissue atlas compared to previously published miRNA expression data from macro-dissection studies of the cortex and medulla of the cat and dog (Ichii et al). Shaded boxes indicate that the dog miRNA was among the top 10 expressed kidney miRNAs in the dog atlas; bolded miRNAs indicate that the miRNA was among the top 20. Comparison between dog whole kidney (dog altas) and data from dog cortex and medulla (Ichii et al) show good correlation with 7/10 miRNAs and 8/10 miRNAs expressed in the top 10 miRNAs, respectively, when compared to kidney expression observed in the dog atlas. Similarly, a comparison of dog whole kidney data (dog atlas) to cat cortex and medulla (Ichii et al) show good correlation as well with 7/10 and 6/10 miRNAs expressed in the top 10 miRNAs, respectively, when compared to dog whole kidney data (dog atlas). (PDF 41 kb)
