# Supplementary material for: The beagle dog MicroRNA tissue atlas: identifying translatable biomarkers of organ toxicity
Source: BMC Genomics. 2016 Aug 17;17:649. doi: 10.1186/s12864-016-2958-x (PMC4989286; doi:10.1186/s12864-016-2958-x)
Supplement: Additional file 1: — Supplementary Methods. (DOCX 18 kb) [file 12864_2016_2958_MOESM1_ESM.docx]

Supplementary **Methods**

### RNA Isolation

#### Tissue

Fifty-micron sections of frozen dog tissue were homogenized with 350 μl lysis buffer (RLT [Qiagen] + 1%-β mercaptoethanol) using a rotor-stator homogenizer and disposable tips. RNA extraction from tissue homogenate was conducted using KingFisher™ Pure RNA Tissue Kit (Thermo Scientific). Briefly, tissue homogenates were combined with magnetic beads and ethanol, and loaded onto a KingFisher Magnetic Particle Processor (Thermo Scientific). Samples were DNase-treated, washed, and eluted in 60 µl of RNase-free water. Due to the low level of total RNA isolated from dog sciatic nerve, the 5 individual samples were pooled and split into 3 sample replicates for analysis. RNA isolation of the bone marrow was performed with 3×volume of TriReagent LS (Life Technologies). After homogenization using a rotor-stator homogenizer, duplicate 1.0-mL aliquots were processed with TRIzol Plus RNA Purification kit (Life Technologies) according to manufacturer instructions. Samples were subjected to on-column DNase treatment, washed, and then eluted in 100 μl of RNase-free water.

#### Plasma and Serum

RNA isolation from dog plasma or serum was performed using 200 μL sample volume for all specimens tested and eluted with 20 μL of water for a final concentration of 10 biofluid equivalents (BE)/μL.

### miR-seq Annotation and Sample Analysis

miRNA reads were normalized on a per sample basis using the total number of reads aligning to miRNAs to derive reads per million (RPM), calculated for each miRNA, *i*, as shown in Equation 1, where *n* is the total number of annotated miRNAs, *c* is number of raw counts, and *M* stands for million [61].

Equation 1:

$$RPM_{i}=\frac{c_{i}}{\sum_{i=1}^{n} c_{i}}* 1 M$$

Where, *RPM_i_* is reads per million, the normalized expression value for miRNA *i*. The term *c_i_* is the number of raw counts for miRNA *i*, *n* is the total number of annotated miRNAs, and 1 M is 1 million. The denominator, $\sum_{i=1}^{n} c_{i}$, is the combined number of raw counts for all annotated miRNAs found in the sample.

#### miRNA Enrichment Criteria

Ubiquitously expressed miRNAs were identified in the dog miRNA tissue atlas by meeting the following criteria: low CV < 10% and standard deviation ≤ 1 in all tissues in the dog atlas.

A standard test was used to define *tissue* *enriched* (TE) based on the level of expression in a tissue versus the rest of tissues in the dog miRNA tissue atlas. For defining *highly tissue enriched* (HTE) miRNAs, a stringency was added by using a modified statistic, *medmax* (Equation 2). Tissue-enriched miRNA were identified and categorized as follows: 1) miRNA that were highly expressed in 1 or 2 tissues relative to the *maximum expression* across all other samples were classified as HTE, and 2) miRNAs that were highly expressed in a given tissue relative to the *average expression* across all other samples were considered TE. By definition, all miRNAs meeting the criteria for HTE also meet the criteria for TE.

Tissue Enriched (TE): An miRNA is considered enriched based on fold change, (average expression in samples within tissue of interest) / (average expression across all other samples) > 5 and a rank-based measure, the FDR-corrected Wilcoxon Rank-Sum p-value < 0.05. In the Wilcoxon Rank-Sum Test, one group was composed of samples within the tissue of interest and the other group was composed of all other samples. The samples were ranked by expression of a given miRNA.

Highly Tissue Enriched (HTE): A miRNA is *highly enriched in a single tissue, T_A_*, if medmax (*T_A_*) > 5 (Equation 2). A miRNA is *highly enriched in 2 tissues*; let *T_A_*_,_ *T_B_*_,_ *T_C_* be the tissues with highest, second-highest, and third-highest median expression for the miRNA, respectively. The miRNA has to be highly expressed at approximately the same level in *T_A_* and *T_B_*, according to median(*T_B_*)/median(*T_A_*) > 70%. The miRNA must be expressed at low levels in all other tissues, according to median(*T_C_*)/median(*T_A_*) < 20%.

Equation 2:

medmax $=\frac{median}{max}$ (median expression of the microRNA in samples belonging to the tissue of interest) / (maximum expression of the microRNA in samples belonging to all other tissues)

For the medmax statistic (Equation 2), the median expression in the tissue of interest divided by the maximum expression across samples was used from all other tissues. Placing the median in the numerator, versus the mean, avoids discarding miRNAs that are highly expressed in most, but not all, samples from the tissue of interest. This acknowledges the fact that expression of a miRNA may vary between samples within a tissue as a result of biological variation (eg., tissue subtypes), but is nonetheless identified as a potential biomarker for damage to the tissue in which it is enriched.

### miRNA Atlas Verification

Q-RT-PCR analysis was conducted on dog atlas tissues to verify tissue expression levels of miRNA candidate biomarkers identified via miRNA-SEQ. Total RNA (1000 ng) was reverse transcribed in a reaction volume of 1520 µL using the Applied Biosystems TaqMan MicroRNA RT Kit High Capacity cDNA Synthesis Kit (Life Technologies) according to manufacturer instructions. 1.33µL from RT reaction 20 ng of total RNA equivalent cDNA (assuming 100% cDNA synthesis efficiency) was mixed with 15 µL Applied Biosystems TaqMan Universal PCR Master Mix, No AmpErase® UNG (Life Technologies) for each Q-RT-PCR reaction in a total volume of 20µL. Following incubation at 50°C for 2 minutes and 95°C for 10 minutes, samples were amplified for 40 cycles (95°C for 15 seconds, then 60°C for 1 minute). All amplifications were performed in triplicate using an Applied Biosystems 7900HT real-time thermocycler. Technical replicate threshold cycle (C_t_) values were averaged for each sample. C_t_ values greater than 38 were flagged because they were within a 10-fold level of the final cycle and were deemed less reliable or not expressed. Relative miRNA expression quantification was derived using the comparative Ct method (Applied Biosystems). The ΔCt (dCt) was calculated by subtracting the average Ct of the ubiquitously expressed miRNAs from Ct value of the individual miRNA of interest. Expression values were calculated using the formula 2^-ΔCt^ × 1000.

## Atlas Tissue, RNA, and Sequencing Quality

Plasma samples collected for the dog tissue atlas had low RIN values (< 2) and were in line with published data. Bone marrow samples had lower percent mapped reads which correlated with low RIN values. However, tissues with low RIN values did not always demonstrate a lower percentage of mapped reads. For example, a subset of kidney samples with RIN values of 1 show similar percent mapping to kidney samples with RIN values > 7. Thymus tissues with RIN values > 7, had the most variable percent mapped reads and miRNAs detected when compared to all other tissues in the atlas. Inter-tissue variation in the thymus could relate to heterogeneity of the tissue samples collected during tissue harvest at the bioreclamation site.
