# Supplementary material for: The beagle dog MicroRNA tissue atlas: identifying translatable biomarkers of organ toxicity
Source: BMC Genomics. 2016 Aug 17;17:649. doi: 10.1186/s12864-016-2958-x (PMC4989286; doi:10.1186/s12864-016-2958-x)
Supplement: Additional file 2: Figure S1. — List of 106 enriched miRNAs by tissue. miRNAs are annotated as tissue enriched (TE) or highly tissue enriched (HTE). The family of miRNA and mature miRNAs in dog, human and rat are given. (PDF 55 kb) [file 12864_2016_2958_MOESM2_ESM.pdf]

Supplemental Figure 1

Tissue	miRNA Family	Mature miRNA in Dog (cfa)	Mature miRNA in Human (hsa)	Mature miRNA in Rat (rno)	Enrichment
Brain	miR-105a	cfa-miR-105a	hsa-miR-105-5p		HTE
Brain	miR-105b	cfa-miR-105b			HTE
Brain	miR-124	cfa-miR-124	hsa-miR-124-3p	rno-miR-124-3p	HTE
Brain	miR-128	cfa-miR-128	hsa-miR-128-3p	rno-miR-128-3p	HTE
Brain	miR-132	cfa-miR-132	hsa-miR-132-3p	rno-miR-132-3p	HTE
Brain	miR-137	cfa-miR-137	hsa-miR-137	rno-miR-137-3p	HTE
Brain	miR-138a	cfa-miR-138a	hsa-miR-138-5p	rno-miR-138-5p	HTE
Brain	miR-149	cfa-miR-149	hsa-miR-149-5p	rno-miR-149-5p	HTE
Brain	miR-212	cfa-miR-212	hsa-miR-212-5p	rno-miR-212-5p	HTE
Brain	miR-219	cfa-miR-219-3p	hsa-miR-219a-2-3p_hsa-miR-219b-5p	rno-miR-219a-2-3p_rno-miR-219b	HTE
Brain	miR-323	cfa-miR-323	hsa-miR-323a-3p	rno-miR-323-3p	HTE
Brain	miR-380	cfa-miR-380			HTE
Brain	miR-410	cfa-miR-410	hsa-miR-410-3p	rno-miR-410-3p	HTE
Brain	miR-487a	cfa-miR-487a	hsa-miR-487a-3p		HTE
Brain	miR-487b	cfa-miR-487b	hsa-miR-487b-3p	rno-miR-487b-3p	HTE
Brain	miR-874	cfa-miR-874	hsa-miR-874-3p	rno-miR-874-3p	HTE
Brain	miR-889	cfa-miR-889	hsa-miR-889-3p		HTE
Brain	miR-9-5p	cfa-miR-9	hsa-miR-9-5p	rno-miR-9a-5p_rno-miR-9b-3p	HTE
Brain	miR-92b	cfa-miR-92b	hsa-miR-92b-3p	rno-miR-92b-3p	HTE
Brain	miR-105		hsa-miR-105-3p		HTE
Brain	miR-128		hsa-miR-128-1-5p	rno-miR-128-1-5p	HTE
Brain	miR-129		hsa-miR-129-2-3p	rno-miR-129-2-3p_rno-miR-129-1-3p	HTE
Brain	miR-1298		hsa-miR-1298-5p	rno-miR-1298	HTE
Brain	miR-132		hsa-miR-132-5p	rno-miR-132-5p	HTE
Brain	miR-136		hsa-miR-136-3p	rno-miR-136-3p	HTE
Brain	miR-139		hsa-miR-139-5p	rno-miR-139-5p	HTE
Brain	miR-212		hsa-miR-212-3p	rno-miR-212-3p	HTE
Brain	miR-431		hsa-miR-431-3p		HTE
Brain	miR-487a		hsa-miR-487a-5p		HTE
Brain	miR-9a-3p		hsa-miR-9a-3p	rno-miR-9b-5p	HTE
Brain, Sciatic Nerve	miR-1249	cfa-miR-1249	hsa-miR-1249-3p	rno-miR-1249	TE
Brain, Sciatic Nerve	miR-127	cfa-miR-127	hsa-miR-127-3p	rno-miR-127-3p	TE
Brain, Sciatic Nerve	miR-184	cfa-miR-184	hsa-miR-184	rno-miR-184	HTE
Brain, Sciatic Nerve	miR-411	cfa-miR-411	hsa-miR-411-5p		TE
Brain, Sciatic Nerve	miR-432	cfa-miR-432	hsa-miR-432-5p		TE
Brain, Sciatic Nerve	miR-504	cfa-miR-504	hsa-miR-504-5p		TE
Brain, Sciatic Nerve	miR-338		hsa-miR-338-5p	rno-miR-338-5p_rno-miR-3065-3p	TE
Brain, Sciatic Nerve	miR-382		hsa-miR-382-5p	rno-miR-382-5p	TE
Brain, Sciatic Nerve	miR-411		hsa-miR-411-3p	rno-miR-411-3p	TE
Brain, Sciatic Nerve	miR-744		hsa-miR-744-5p		TE
Brain, Testis	miR-146b	cfa-miR-146b	hsa-miR-146b-5p	rno-miR-146b-5p	TE
Brain, Testis	miR-335	cfa-miR-335	hsa-miR-335-5p	rno-miR-335	TE
Brain, Testis	miR-335		hsa-miR-335-3p		TE
Brain, Testis	miR-873		hsa-miR-873-5p	rno-miR-873-5p	TE
Brain, Jejunum	miR-342	cfa-miR-342	hsa-miR-342-3p	rno-miR-342-3p	TE
Brain, Liver	miR-885	cfa-miR-885	hsa-miR-885-5p		TE
Brain, Pancreas	miR-135a	cfa-miR-135a-5p	hsa-miR-135a-5p	rno-miR-135a-5p	TE
Brain, Pancreas	miR-153	cfa-miR-153	hsa-miR-153-3p	rno-miR-153-3p	TE
Bone Marrow	miR-8865	cfa-miR-8865			HTE
Bone Marrow, Brain	miR-129	cfa-miR-129	hsa-miR-129-5p	rno-miR-129-5p	TE
Bone Marrow, Brain	miR-628	cfa-miR-628	hsa-miR-628-5p		TE
Bone Marrow, Brain	miR-7	cfa-miR-7	hsa-miR-3529-3p	rno-miR-7a-5p	HTE
Bone Marrow, Brain	miR-129-1		hsa-miR-129-1-3p	rno-miR-129-1-3p	TE
Bone Marrow, Jejunum	miR-374b	cfa-miR-374b	hsa-miR-374b-5p_hsa-miR-374c-3p	rno-miR-374-5p_rev	TE
Bone Marrow, Plasma	miR-144	cfa-miR-144	hsa-miR-144-3p	rno-miR-144-3p	TE
Bone Marrow, Plasma	miR-25	cfa-miR-25	hsa-miR-25-3p	rno-miR-25-3p	TE
Bone Marrow, Plasma	miR-451	cfa-miR-451	hsa-miR-451a	rno-miR-451-5p	TE
Bone Marrow, Plasma	miR-144		hsa-miR-144-5p	rno-miR-144-5p	HTE
Bone Marrow, Testis	miR-450a	cfa-miR-450a	hsa-miR-450a-5p	rno-miR-450b-5p	TE
Bone Marrow, Testis	miR-450b	cfa-miR-450b	hsa-miR-450b-5p		TE
Colon, Ileum	miR-147	cfa-miR-147	hsa-miR-147b	rno-miR-147	TE
Heart	miR-499	cfa-miR-499	hsa-miR-499a-5p	rno-miR-499-5p	HTE
Heart, Striated Muscle	miR-1	cfa-miR-1	hsa-miR-1-3p	rno-miR-1b	TE
Heart, Striated Muscle	miR-133a	cfa-miR-133a	hsa-miR-133a-3p	rno-miR-133c_rno-miR-133a-3p	HTE
Heart, Striated Muscle	miR-133b	cfa-miR-133b	hsa-miR-133b	rno-miR-133b-3p	TE
Heart, Striated Muscle	miR-208b	cfa-miR-208b	hsa-miR-208b-3p	rno-miR-208b-3p	TE
Heart, Striated Muscle	miR-133a		hsa-miR-133a-5p	rno-miR-133a-5p	HTE
Ileum, Jejunum	miR-215	cfa-miR-215			TE
Liver	miR-122	cfa-miR-122	hsa-miR-122-5p_hsa-miR-3591-3p	rno-miR-122-5p_rno-miR-3591-3p	HTE
Liver	miR-3591		hsa-miR-122-3p_hsa-miR-3591-5p		HTE
Liver, Pancreas	miR-148a-5p		hsa-miR-148a-5p		TE
Pancreas	miR-148a-3p	cfa-miR-148a	hsa-miR-148a-3p	rno-miR-148a-3p	HTE
Pancreas	miR-216a	cfa-miR-216a	hsa-miR-216a-5p	rno-miR-216a-5p	HTE
Pancreas	miR-216b	cfa-miR-216b	hsa-miR-216b-5p	rno-miR-216b-5p	HTE
Pancreas	miR-217	cfa-miR-217	hsa-miR-217		HTE
Pancreas	miR-375	cfa-miR-375	hsa-miR-375	rno-miR-375-3p	HTE
Pancreas	miR-216a			rno-miR-216a-3p	HTE
Pancreas	miR-217			rno-miR-217-3p	HTE
Pancreas, Jejunum	miR-141	cfa-miR-141	hsa-miR-141-3p	rno-miR-141-3p	TE
Pancreas, Jejunum	miR-802	cfa-miR-802	hsa-miR-802	rno-miR-802-5p	TE
Plasma	miR-423a	cfa-miR-423a	hsa-miR-423-5p_rev_hsa-miR-3184-3p	rno-miR-423-5p	HTE
Plasma	miR-16			rno-miR-16-3p	HTE
Plasma, Brain	miR-107	cfa-miR-107	hsa-miR-107	rno-miR-107-3p	TE
Plasma, Brain	miR-383	cfa-miR-383	hsa-miR-383-5p		TE
Plasma, Brain	miR-331		hsa-miR-331-5p		TE
Plasma, Ileum	miR-15a	cfa-miR-15a	hsa-miR-15a-5p	rno-miR-15a-5p	TE
Sciatic Nerve, Thymus	miR-193a	cfa-miR-193a	hsa-miR-193a-5p		TE
Striated Muscle	miR-206	cfa-miR-206	hsa-miR-206	rno-miR-206-3p	HTE
Testis	miR-202	cfa-miR-202	hsa-miR-202-5p	rno-miR-202-5p	HTE
Testis	miR-34b	cfa-miR-34b		rno-miR-34b-5p	HTE
Testis	miR-34c	cfa-miR-34	hsa-miR-34c-5p	rno-miR-34c-5p	HTE
Testis	miR-449a	cfa-miR-449a	hsa-miR-449a	rno-miR-449a-5p	HTE
Testis	miR-506	cfa-miR-506			HTE
Testis	miR-507b	cfa-miR-507b			HTE
Testis	miR-508a	cfa-miR-508a			HTE
Testis	miR-508b	cfa-miR-508b			HTE
Testis	miR-8831	cfa-miR-8831			HTE
Testis	miR-8908a-3p	cfa-miR-8908a-3p			HTE
Testis	miR-8908a-5p	cfa-miR-8908a-5p			HTE
Testis	miR-8908b	cfa-miR-8908b			HTE
Testis	miR-8908c	cfa-miR-8908c			HTE
Testis	miR-34b		hsa-miR-34b-3p	rno-miR-34b-3p	HTE
Testis	miR-34c		hsa-miR-34c-3p		HTE
Testis	miR-449b		hsa-miR-449b-5p		HTE
Testis, Thymus	miR-106a	cfa-miR-106a	hsa-miR-106a-5p		TE
Testis, Thymus	miR-205	cfa-miR-205	hsa-miR-205-5p	rno-miR-205	HTE
