# Supplementary material for: The beagle dog MicroRNA tissue atlas: identifying translatable biomarkers of organ toxicity
Source: BMC Genomics. 2016 Aug 17;17:649. doi: 10.1186/s12864-016-2958-x (PMC4989286; doi:10.1186/s12864-016-2958-x)
Supplement: Additional file 3: Figure S2. — Top 20 enriched miRNAs. 10 of top 20 are testis. (PDF 38 kb) [file 12864_2016_2958_MOESM3_ESM.pdf]

Supplemental Figure 2

Top 20 Enriched	Tissue	miRNA	med_over _max	Wilcox p-value	Median (RPM)
1	Testis	cfa-miR-508b	581	2.41E-11	660
2	Testis	cfa-miR-202_hsa-miR-202-5p_rno-miR-202-5p	420	3.30E-05	3340
3	Brain	cfa-miR-124_hsa-miR-124-3p_rno-miR-124-3p	347	6.84E-05	2971
4	Pancreas	cfa-miR-217_hsa-miR-217	295	4.50E-05	3543
5	Pancreas	cfa-miR-216b_hsa-miR-216b-5p_rno-miR-216b-5p	275	6.53E-05	5507
6	Testis	cfa-miR-8831	260	2.54E-10	268
7	Testis	cfa-miR-8908b	222	2.83E-07	456
8	Testis	cfa-miR-508a	212	1.17E-10	153
9	Testis	cfa-miR-506	185	4.75E-64	1094
10	Liver	hsa-miR-122-3p_hsa-miR-3591-5p	184	1.12E-05	838
11	Liver	cfa-miR-122_hsa-miR-122-5p_rno-miR-122-5p_hsa-miR-3591-3p	183	9.07E-05	22008
12	Testis	cfa-miR-8908c	132	9.38E-09	271
13	Brain	cfa-miR-219-3p_hsa-miR-219b-5p_rno-miR-219b	101	7.43E-07	276
14	Brain	hsa-miR-9-3p_rno-miR-9a-3p_rno-miR-9b-5p	97	3.81E-05	527
15	Testis	cfa-miR-507b	86	1.68E-06	101
16	Pancreas	cfa-miR-216a_hsa-miR-216a-5p_rno-miR-216a-5p	81	7.43E-05	4143
17	Pancreas	rno-miR-217-3p	77	4.71E-07	349
18	Testis	cfa-miR-449a_hsa-m1R-449a_rno-miR-449a-5p	73	1.07E-64	3159
19	Testis	cfa-miR-507a	69	2.25E-12	34
20	Brain	cfa-miR-9_hsa-m1R-9-5p_rno-miR-9a-5p_rno-miR-9b-3p	61	1.03E-64	38121
