# Supplementary material for: The beagle dog MicroRNA tissue atlas: identifying translatable biomarkers of organ toxicity
Source: BMC Genomics. 2016 Aug 17;17:649. doi: 10.1186/s12864-016-2958-x (PMC4989286; doi:10.1186/s12864-016-2958-x)
Supplement: Additional file 4: Figure S3. — Top Ten Expressed (log10 RPM values) miRNA per tissue type. The list is sorted by dog miRNA tissue atlas enrichment and prevalence of top 10 expressed miRNAs across the atlas tissues. (PDF 45 kb) [file 12864_2016_2958_MOESM4_ESM.pdf]

Supplemental Figure 3

Dog microRNA Annotation (cfa)	Enrichment	#Top 10 in tissue	Bone Marrow	Brain	Colon	Duodenum	Heart	Ileum	Jejunum	Kidney	Liver	Lung	Muscle	Pancreas	Plasma	Sciatic Nerve	Testis	Thymus
cfa-miR-128_hsa-miR-128-3p_rno-miR-128-3p	Yes	1		4.5														
cfa-miR-215	Yes	1							4.7									
cfa-miR-34c_hsa-miR-34c-5p_rno-miR-34c-5p	Yes	1															4.7	
cfa-miR-375_hsa-miR-375_rno-miR-375-3p	Yes	1												5.2				
cfa-miR-451_hsa-miR-451a_rno-miR-451-5p	Yes	1	4.6															
cfa-miR-92b_hsa-miR-92b-3p_rno-miR-92b-3p	Yes	1		4.4														
cfa-miR-133a_hsa-miR-133a-3p_rno-miR-133a-3p	Yes	2					4.9						4.8					
cfa-miR-148a_hsa-miR-148a-3p_rno-miR-148a-3p	Yes	2									4.8			5.6				
cfa-miR-451_hsa-miR-451a_rno-miR-451-5p	No	2													4.9			
cfa-miR-25_hsa-miR-25-3p_rno-miR-25-3p	No	1													4.4			
cfa-miR-10b_hsa-miR-10b-5p_rno-miR-10b-5p	No	1								5.7								
cfa-miR-127_hsa-miR-127-3p_rno-miR-127-3p	No	1		4.7														
cfa-miR-142_hsa-miR-142-5p_rno-miR-142-5p	No	1													4.4			
cfa-miR-16_hsa-miR-16-5p_rno-miR-16-5p	No	1													4.6			
cfa-miR-194_hsa-miR-194-5p_rno-miR-194-5p	No	1							4.5									
cfa-miR-30d_hsa-miR-30d-5p_rno-miR-30d-5p	No	1					4.6											
cfa-miR-423a_hsa-miR-423-5p_rev__rno-miR-423-5p	No	1													4.3			
cfa-miR-486_hsa-miR-486-3p_rno-miR-486	No	1													5.8			
cfa-miR-9_hsa-miR-9-5p_rno-miR-9a-5p	No	1		4.6														
cfa-miR-93_hsa-miR-93-5p_rno-miR-93-5p	No	1													4.2			
cfa-miR-186_hsa-miR-186-5p_rno-miR-186-5p	No	2	4.3												4.1			
cfa-miR-92a_hsa-miR-92a-3p_rno-miR-92a-3p	No	2													4.5		4.3	
cfa-let-7a_hsa-let-7a-5p_rno-let-7a-5p_rev	No	3		4.6								4.6				4.6		
cfa-miR-21_hsa-miR-21-5p_rno-miR-21-5p	No	3			4.6			4.8	4.5									
cfa-miR-126_hsa-miR-126-5p_rno-miR-126a-5p	No	4	4.3				4.6					4.7						4.4
cfa-let-7f_hsa-let-7f-5p_rno-let-7f-5p_rno-miR-3596d	No	6						4.4			4.6	4.5		4.6		4.4	4.4	
cfa-miR-192_hsa-miR-192-5p_rno-miR-192-5p	No	7			5.2	4.9		5.2	5.5	4.7	5.3			4.4				
cfa-miR-486_hsa-miR-486-3p_rno-miR-486	No	7	4.8			4.3	5.1					4.6	4.9			4.9		4.6
cfa-miR-22_hsa-miR-22-3p_rno-miR-22-3p	No	8		4.5			5.0			4.6	5.1		4.8	4.6		4.5	4.4	
cfa-miR-30a_hsa-miR-30a-5p_rno-miR-30a-5p	No	8	4.3				4.7			4.9	4.6	4.9	4.5	4.4				4.6
cfa-miR-378_hsa-miR-378a-3p_rev_rno-miR-378a-3p	No	8			4.3	4.3	4.8		4.4	4.2	4.5		4.8					4.7
cfa-miR-27b_hsa-miR-27b-3p_rno-miR-27b-3p	No	9			4.4	4.4	4.7	4.4	4.3			4.6	4.5	4.2		4.5		
cfa-miR-191_hsa-miR-191-5p_rno-miR-191a-5p	No	10	4.5	4.6	4.4	4.3		4.6		4.2					4.4	4.5	4.3	4.5
cfa-miR-10a_hsa-miR-10a-5p_rno-miR-10a-5p	No	11	4.9		4.7	4.5		4.8	4.8	5.0	4.5	5.0	4.4				4.5	4.8
cfa-miR-10b_rno-miR-10b-5p_hsa-miR-10b-5p	No	11	5.5		4.9	4.7	4.9	4.9	4.4				5.6	4.3		5.5	5.6	5.4
cfa-miR-181a_hsa-miR-181a-5p_rno-miR-181a-5p	No	12	4.5	5.3	4.3	4.3		4.4		4.6	4.5	5.0		4.3		4.8	4.4	4.7
cfa-miR-143_hsa-miR-143-3p	No	13		4.5	5.6	5.3	5.0	5.4	5.2	4.3	4.8	5.1	4.5			4.8	5.0	5.0
cfa-miR-26a_hsa-miR-26a-5p_rno-miR-26a-5p	No	14	5.1	4.7	4.4	4.5		4.3	4.6	4.5	4.5	4.9	4.5	4.6		4.6	4.3	4.4
