# Supplementary material for: The beagle dog MicroRNA tissue atlas: identifying translatable biomarkers of organ toxicity
Source: BMC Genomics. 2016 Aug 17;17:649. doi: 10.1186/s12864-016-2958-x (PMC4989286; doi:10.1186/s12864-016-2958-x)

Supplemental Figure 4

Supplemental Figure XXX: Benefit and need for enhanced annotations for miRNA studies

A

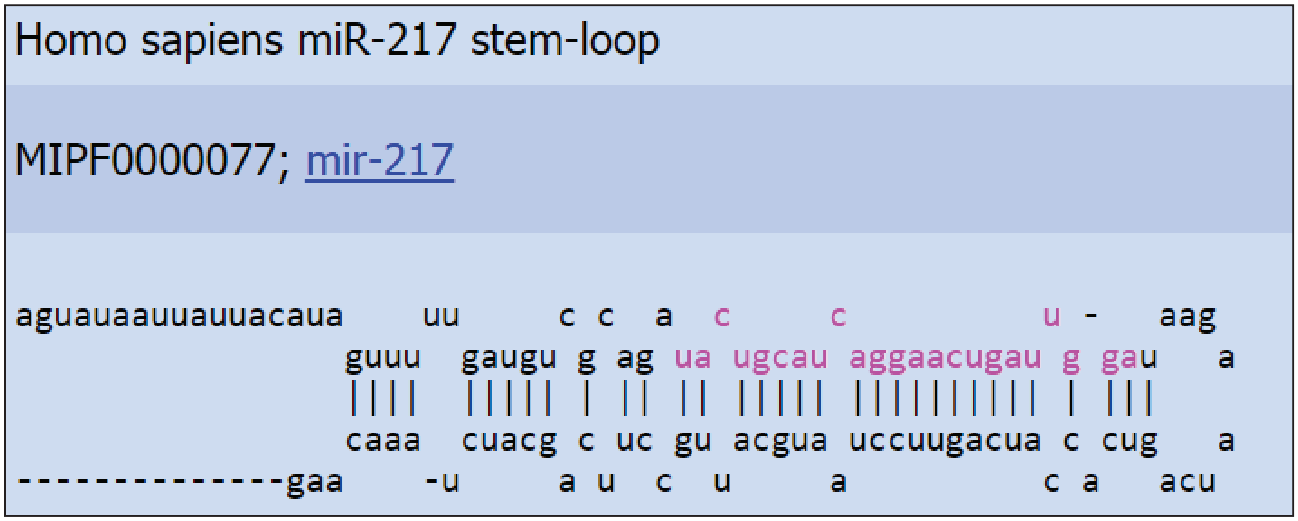

B

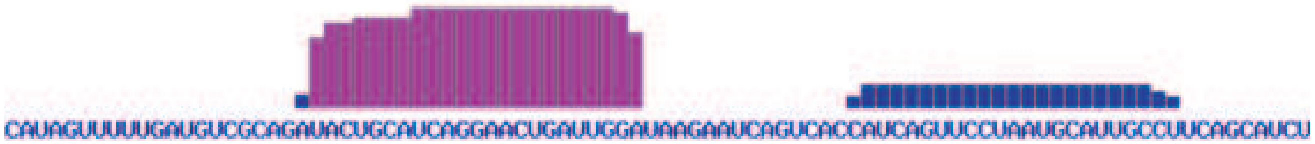

Supplement: Additional file 5: Figure S4. — Benefit and need for enhanced annotations for miRNA studies. (A) Pancreas enriched miR-217-3p (rno-miR-217-3p AUCAGUUCCUAAUGCAUUGCCU) identified in the dog miRNA tissue atlas was found as a novel un-annotated human miRNA. It is conserved in rats, dogs, and humans, but is only annotated in miRBase as rat. (B) There is evidence of un-annotated human reads aligning and expressed in human expression profile experiments on the miRBase website. This study suggests human miRBase annotation should reflect 2 mature microRNA sequences, miR-3p and -5p. (PDF 64 kb) [file 12864_2016_2958_MOESM5_ESM.pdf]
