# Supplementary material for: The beagle dog MicroRNA tissue atlas: identifying translatable biomarkers of organ toxicity
Source: BMC Genomics. 2016 Aug 17;17:649. doi: 10.1186/s12864-016-2958-x (PMC4989286; doi:10.1186/s12864-016-2958-x)
Supplement: Additional file 6: Figure S5. — Q-RT-PCR assay list. (PDF 39 kb) [file 12864_2016_2958_MOESM6_ESM.pdf]

Supplemental Figure 5

Dog miRNA Name	Assay Name	Assay ID*	miRBase acc. #
cfa-miR-1	hsa-miR-1	00385	MI0018528
cfa-miR-122	hsa-miR-122	002245	MI0000256
cfa-miR-133a	hsa-miR-133a	000458	MI0000362
cfa-miR-133b	hsa-miR-133b	002247	MI0000821
cfa-miR-16	hsa-miR-16	000391	MI0000565
cfa-miR-186	hsa-miR-186	002285	MI0000228
cfa-miR-192	hsa-miR-192	000491	MI0000551
cfa-miR-193a	hsa-miR-193a-5p	002281	MI0005014
cfa-miR-193b	hsa-miR-193b*	002366	MI0005484
cfa-miR-200a	hsa-miR-2013a*	001011	MI0000554
cfa-miR-206	hsa-miR-206	000510	MI0000249
cfa-miR-208b	hsa-miR-208b	002290	MI0005552
cfa-miR-21	hsa-miR-21	000397	MI0000569
cfa-miR-212	mmu-miR-212-5p	461768_mat	MI0000696
cfa-miR-216a	hsa-miR-216	000519	MI0010344
cfa-miR-216b	hsa-miR-216b	002326	MI0004126
cfa-miR-29a	hsa-miR-29a	002112	MI0000576
cfa-miR-34b	bta-miR-34b	007904_mat	MI0004763
cfa-miR-34c	hsa-miR-34c	000428	MI0000403
cfa-miR-432	hsa-miR-432	001026	MI0009831
cfa-miR-499	mmu-miR-499	001352	MI0004676
cfa-miR-885	hsa-miR-885-5p	002296	MI0009904

*Applied Biosystems
Assay Name
