# Supplementary material for: The beagle dog MicroRNA tissue atlas: identifying translatable biomarkers of organ toxicity
Source: BMC Genomics. 2016 Aug 17;17:649. doi: 10.1186/s12864-016-2958-x (PMC4989286; doi:10.1186/s12864-016-2958-x)
Supplement: Additional file 7: Figure S6. — Top 20 expressed kidney miRNA. Top 20 expressed kidney miRNA found in the dog miRNA tissue atlas compared to previously published miRNA expression data from macro-dissection studies of the cortex and medulla of the cat and dog (Ichii et al). Shaded boxes indicate that the dog miRNA was among the top 10 expressed kidney miRNAs in the dog atlas; bolded miRNAs indicate that the miRNA was among the top 20. Comparison between dog whole kidney (dog altas) and data from dog cortex and medulla (Ichii et al) show good correlation with 7/10 miRNAs and 8/10 miRNAs expressed in the top 10 miRNAs, respectively, when compared to kidney expression observed in the dog atlas. Similarly, a comparison of dog whole kidney data (dog atlas) to cat cortex and medulla (Ichii et al) show good correlation as well with 7/10 and 6/10 miRNAs expressed in the top 10 miRNAs, respectively, when compared to dog whole kidney data (dog atlas). (PDF 41 kb) [file 12864_2016_2958_MOESM7_ESM.pdf]

Supplemental Figure 6

Dog miRNA Atlas	Ichii et al (Dog)		Ichii et al (Cat)	
Whole Kidney	Cortex	Medulla	Cortex	Medulla
cfa-miR-10b	miR-10b-5p	miR-26a-5p	miR-22-3p	miR-26a-5p
cfa-miR-10a	miR-192-5p	miR-10b-5p	miR-192-5p	miR-10b-5p
cfa-miR-30a	miR-26a-5p	miR-10a-5p	miR-27b-3p	miR-181a-5p
cfa-miR-192	miR-22-3p	miR-181a-5p	**miR-204-5p**	miR-10a-5p
cfa-miR-22	miR-191-5p	miR-22-3p	miR-181a-5p	miR-22-3p
cfa-miR-181a	**miR-204-5p**	miR-192-5p	miR-10b-5p	**miR-27b-3p**
cfa-miR-26a	miR-181a-5p	miR-191-5p	miR-99b-5p	miR-99b-5p
cfa-miR-143	**let-7f-5p**	**miR-126-5p**	miR-191-5p	miR-191-5p
cfa-miR-191	miR-10a-5p	**miR-27b-3p**	miR-30a-3p	miR-30a-5p
cfa-miR-378	**miR-27b-3p**	miR-30a-5p	miR-126-5p	miR-126-5p
**cfa-let-7a**	miR-126-5p	**let-7f-5p**	miR-10a-5p	**let-7f-5p**
**cfa-let-7f**	miR-30a-5p	**let-7a-5p**	**miR-26a-5p**	**let-7a-5p**
**cfa-miR-186**	**let-7a-5p**	miR-99b-5p	**miR-186-5p**	miR-100-5p
**cfa-miR-27b**	**miR-186-5p**	miR-99b-5p	miR-320-3p	miR-186-5p
**cfa-miR-30d**	miR-99b-5p	**miR-204-5p**	let-7b-5p	**miR-30c-5p**
**cfa-miR-204**	miR-16-5p	**miR-30c-5p**	miR-100-5p	miR-125a-5p
**cfa-miR-30c**	let-7c-5p	let-7g-5p	miR-99a-5p	miR-16-5p
**cfa-miR-126**	**miR-30c-5p**	miR-16-5p	miR-181b-5p	**miR-204-5p**
**cfa-miR-486**	let-7g-5p	let-7b-5p	miR-103-3p	let-7g-5p
**cfa-miR-30e-5p**	miR-103-3p	miR-30a-3p	miR-193b-3p	miR-125b-5p
